# Expression of Cyclin D1, p53, and Tumor-Associated Tissue Eosinophils in Different Grades of Oral Squamous Cell Carcinoma

**DOI:** 10.7759/cureus.99291

**Published:** 2025-12-15

**Authors:** Momina Habib, Manal Rauf, Sidra Omair, Summaya S Chaudry, Abid R Khan, Ahson Ahmad, Ahmareen Sheikh

**Affiliations:** 1 Histopathology, Pakistan Institute of Medical Sciences, Islamabad, PAK; 2 Histopathology, Shifa College of Medicine, Islamabad, PAK; 3 Histopathology, Shaheed Zulfiqar Ali Bhutto Medical University, Pakistan Institute of Medical Sciences, Islamabad, PAK; 4 Otolaryngology, Pakistan Institute of Medical Sciences, Islamabad, PAK; 5 Radiology, Shifa International Hospital, Islamabad, PAK; 6 Pathology, Pakistan Institute of Medical Sciences, Islamabad, PAK

**Keywords:** cyclin d1, grades, histological grades, oral cavity cancers, p53, prognosis, squamous cell carcinoma, tate

## Abstract

Background

Oral squamous cell carcinoma (SCC) is a significant health burden, particularly in developing countries. Cyclin D1 and p53 regulate cell cycle progression and apoptosis, while tumor-associated tissue eosinophilia (TATE) influences the tumor microenvironment. In this study, we aimed to assess the correlation between cyclin D1, p53, and TATE expression and tumor grade and histological subtypes in oral SCC.

Methodology

A total of 90 SCC patients were studied at the Pakistan Institute of Medical Sciences Hospital over one year. Tumors were categorized into well-differentiated, moderately differentiated, poorly differentiated, and non-keratinizing subtypes. Immunohistochemical staining for cyclin D1 and p53 was graded (0-3), while TATE was classified as grade 1 (0-10), grade 2 (11-20), and grade 3 (>20 eosinophils per high-power field). Statistical analyses were performed using SPSS version 21 (IBM Corp., Armonk, NY, USA).

Results

Cyclin D1 and p53 expression significantly increased with tumor grade (p = 0.001), whereas TATE was more prevalent in lower-grade SCC (p = 0.03). Non-keratinizing SCC showed the highest cyclin D1 (66.6%) and p53 (100%) expression, whereas 66% had grade 3 TATE.

Conclusions

Cyclin D1 and p53 are potential prognostic markers for SCC, whereas TATE may influence tumor differentiation.

## Introduction

Oral squamous cell carcinoma (OSCC) is recognized as a heterogeneous cancer arising from the lining of the oral cavity mucosa. OSCC accounts for 90% of head and neck squamous cell carcinoma (HNSCC) cases arising from several sites within the oral cavity. It is the most common type of HNSCC, with approximately more than 350,000 new cases reported annually. It is particularly prevalent in developing countries in specific geographic regions. Globally, one-third of these cases are reported from Southeast Asia [1]. In this region, OSCC is the most prevalent cancer in India, Pakistan, Bangladesh, and Sri Lanka. OSCC is the second leading cause of cancer-related deaths among females in Pakistan [2]. Major risk factors for OSCC include tobacco use, alcohol consumption, and human papillomavirus infection. Despite considerable advancements in treatment, the high recurrence rate poses a significant challenge, leading to poor survival outcomes [3].

Among its morphological features, tumor-associated tissue eosinophilia (TATE) has garnered increasing interest in recent years. Eosinophils, a minor subset of leukocytes comprising 1-3% of circulating white blood cells, are linked to various diseases; however, their role in cancer pathophysiology remains controversial. Specifically, the relationship between TATE and oral cancer development is uncertain and contradictory [4]. The total eosinophil count per 10 high-power field (HPF) has been reported to be significantly higher in OSCC than in healthy tissue. Furthermore, the mean TATE score is markedly increased in OSCC tissue. However, the variation in eosinophil counts across different OSCC grades remains unclear [5].

Cyclin D1 regulates the G1-S phase transition through its interaction with CDK4/6, forming the cyclin D1-CDK4/6 complex that phosphorylates the retinoblastoma protein (pRb). Hyperphosphorylated pRb releases E2F transcription factors, promoting cell cycle progression and uncontrolled proliferation in cancers, including OSCC. Therefore, overexpression of cyclin D1 disrupts normal checkpoint control, facilitating tumor aggressiveness and poor differentiation [6,7].

Similarly, p53 maintains genomic integrity through cell-cycle arrest, DNA repair, and apoptosis. Mutant p53 loses tumor-suppressive activity and becomes resistant to MDM2-mediated ubiquitination and degradation. This leads to intracellular accumulation detectable by immunohistochemistry, reflecting dysregulated p53-MDM2 feedback loops and contributing to oncogenesis, therapy resistance, and poorer prognosis [8-10].

This study aimed to assess the correlation of cyclin D1, p53, and TATE expression with tumor grade and histological subtypes in OSCC. By correlating these molecular and inflammatory markers with tumor differentiation and histological subtypes, this study seeks to enhance the understanding of OSCC pathogenesis, refine prognostic models, and potentially identify biomarkers for personalized treatment strategies.

## Materials and methods

This descriptive, cross-sectional study was conducted at the Pakistan Institute of Medical Sciences, Islamabad, from January 2024 to December 2024. After obtaining approval from the ethical committee of the hospital (approval number: F-5-2/2024 (ERCC)/PIMS), oral mucosal biopsies and surgical resection specimens of 90 patients diagnosed with OSCC in the Department of Histopathology were selected. Patients with metastatic carcinomas to the oral cavity with histopathological diagnoses other than OSCC were excluded from the study. All specimens were fixed in 10% formalin, followed by gross examination, sectioning, embedding in paraffin blocks, cutting, slide preparation, and staining of the tissue with eosin and hematoxylin. The slides were examined under a light microscope by two consultant histopathologists. OSCC was subclassified into different histological subtypes and was graded as well-differentiated, moderately differentiated, poorly differentiated, and non-keratinizing squamous cell carcinoma (SCC). Immunohistochemistry was applied for p53 and cyclin D1 expression on duplicate slides for each case. The slides were deparaffinized, rehydrated, and then subjected to antigen retrieval. The reaction was followed by incubation of p53 as well as cyclin D1 monoclonal antibody separately for both slides. The antigen-antibody complexes were visualized using an advanced detection system (Dako), and after various steps of slide preparation, nuclear and cytoplasmic brown staining was taken as a positive result. Immunohistochemical interpretation of cyclin D1 and p53 was performed by two qualified histopathologists based on expression and intensity, recorded on a semi-quantitative scale. Scoring of cyclin D1 was done as shown in Table 1, and that of p53 was done as shown in Table 2.

**Table 1:** Scoring of cyclin D1 expression. Adapted from Anjum et al. [11].

Labeling index (LI)	Intensity (I)	Expression score (LI × I)	Cyclin D1 interpretation
≤10% positive cells - 0	Nil - 0	0	Negative
11–30% positive cells - 1	Faint - 1		
31–50% positive cells - 2	Moderate - 2	1–12	Positive
≥50% positive cells - 3	Intense - 3		

**Table 2:** Scoring of p53 expression. Adapted from Ghanghoria et al. [12].

P53 staining score	Percentage of positive cells	Interpretation
+++	Greater than 50% positive cells	Strong positive staining
++	26% to 50% positive cells	Moderate positive staining
+	5% to 25% positive cells	Weak positive staining
-	Fewer than 5% positive cells	Negative

Similarly, TATE gradient was noted as 0-10, 11-20, and >20 eosinophils per 10 HPF and was categorized as grades 1, 2, and 3, respectively, as shown in Table 3 [13].

**Table 3:** TATE gradient scoring. Adapted from Sharma et al. [13]. TATE = tumor-associated tissue eosinophilia; HPF = high-power field

TATE grade	Eosinophils per HPF	Interpretation
Grade 1	0–10 eosinophils/HPF	Mild
Grade 2	11–20 eosinophils/HPF	Moderate
Grade 3	More than 20 eosinophils/HPF	Severe

The collected data were analyzed using SPSS version 21 (IBM Corp., Armonk, NY, USA). The association between p53, cyclin D1 expression, and TATE with histological grade and subtype of OSCC was assessed using the chi-square test. A p-value ≤0.05 was considered statistically significant.

## Results

Of the 90 patients, 60% (54/90) were males, while 40% (36/90) were females. The age of the patients ranged from 25 to 90 years. Regarding the frequency of grades, the most common was well-differentiated, accounting for 46% (41/90) of cases, followed by moderately differentiated cases at 34% (31/90) and poorly differentiated cases at 17% (15/90). The least common grade was poorly differentiated OSCC, accounting for only 3% (3/90) of cases. Regarding histological subtypes, conventional OSCC was the most common, accounting for 71% of cases, while the least common subtype was adenosquamous, followed by acantholytic (Table 4).

**Table 4:** Frequency of different histological subtypes of OSCC. Conventional OSCC was the most prevalent subtype, followed by basaloid, adenosquamous, verrucous, and acantholytic types. OSCC = oral squamous cell carcinoma

Subtype	Percent
Conventional	70%
Basaloid	15%
Verrucous	10%
Adenosquamous	3%
Acantholytic	2%

Cyclin D1 expression was found in 61% (55/90) of cases. The expression of this immunomarker varied among different histological subtypes of OSCC. Cyclin D1 expression was observed in 50% of acantholytic OSCC (1/2 cases), 41% of conventional OSCC (26/64 cases), and 38% of the basaloid subtype (8/13 cases). In contrast, 0% of verrucous OSCC (0/3 cases) and adenosquamous OSCC (0/8 cases) exhibited cyclin D1 positivity. This difference was statistically significant, with a p-value of 0.001. Similarly, the association between cyclin D1 expression and tumor grade was found to be statistically significant (p = 0.001). Expression of cyclin D1 increased as the tumor grade increased from 9.7% in well-differentiated and 66.6% in non-keratinizing ones (Table 5).

**Table 5:** Cyclin D1 expression across histological grades of OSCC. OSCC = oral squamous cell carcinoma; SCC = squamous cell carcinoma

Histological type	Positive (n)	Negative (n)	Total (n)
Well-differentiated SCC	4	37	41
Moderately differentiated SCC	15	16	31
Poorly differentiated SCC	14	1	15
Non-keratinizing	2	1	3

**Figure 1:**
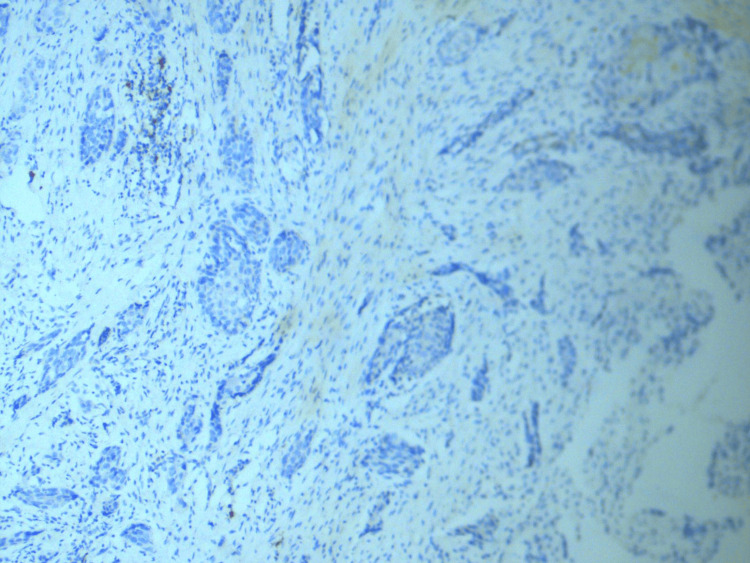
<10% expression of cyclin D1 in well-differentiated OSCC (immunohistochemical stain at 40× magnification). The image shows minimal nuclear cyclin D1 staining in a well-differentiated OSCC, indicative of less expression of cyclin D1 in low tumor grades. OSCC = oral squamous cell carcinoma

**Figure 2:**
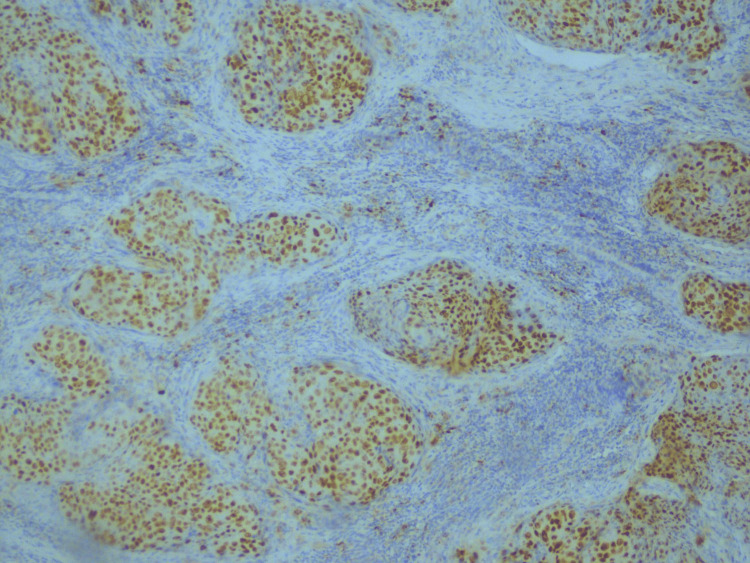
Strong positive expression of cyclin D1 in poorly differentiated OSCC (immunohistochemical stain at 40× magnification). The image demonstrates intense nuclear staining for cyclin D1 in tumor cells, signifying high expression of cyclin D1 as the tumor grade increases. OSCC = oral squamous cell carcinoma

Well-differentiated cases of SCC showed less than 10% staining of cyclin D1, as shown in Figure 1, whereas as the tumor grade increased to poorly differentiated SCC, cyclin D1 expression also increased (Figure 2).

**Figure 3:**
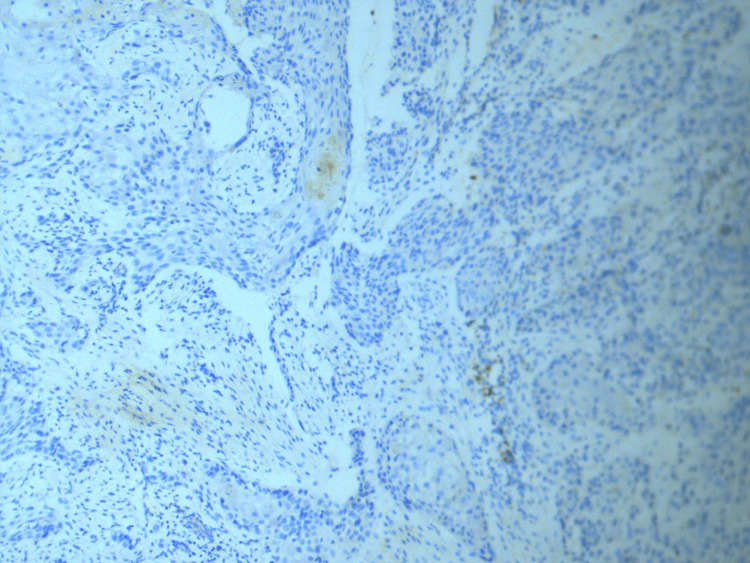
Weak expression pf p53 in well-differentiated OSCC (immunohistochemical stain at 40× magnification). Weak nuclear staining is evident for p53 in well-differentiated OSCC, correlating with low expression of p53 in lower tumor grade. OSCC = oral squamous cell carcinoma

**Figure 4:**
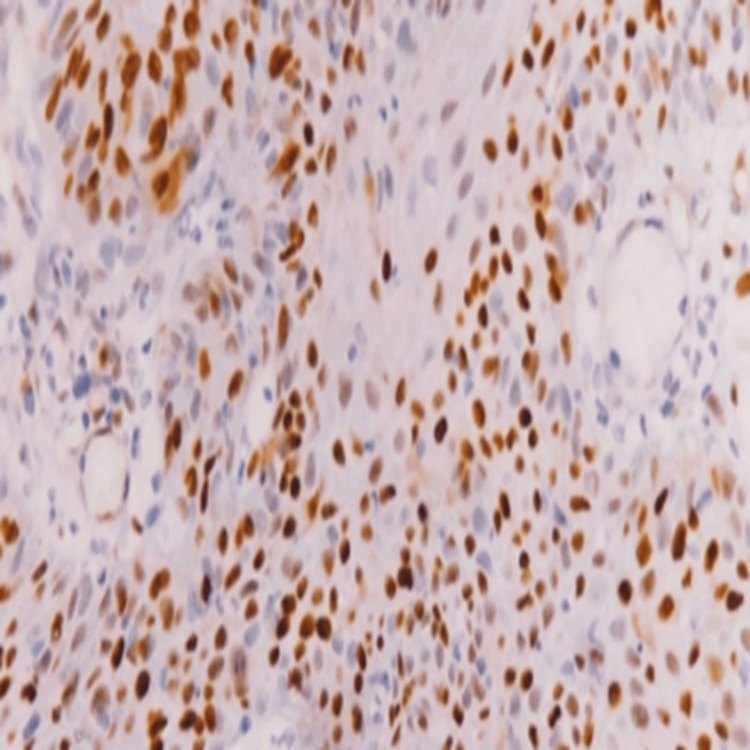
Strong expression of p53 in poorly differentiated OSCC (immunohistochemical stain at 40× magnification). Nuclear overexpression of p53 is clearly visible in this poorly differentiated OSCC, indicating accumulation due to possible TP53 mutation. OSCC = oral squamous cell carcinoma

**Figure 5:**
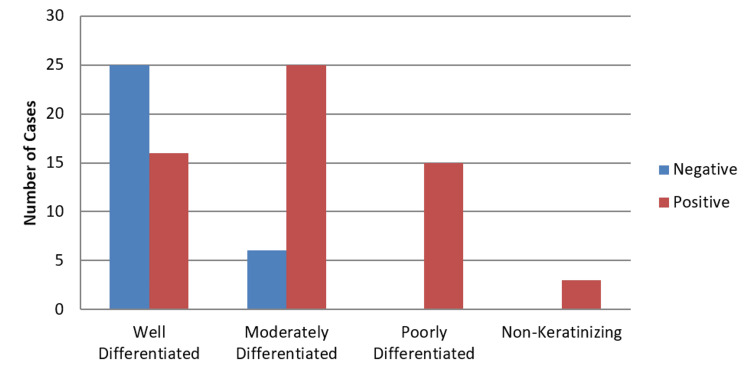
Association of p53 with grades of OSCC. The graph demonstrates a rising trend of p53 positivity with increasing tumor grade. All poorly differentiated and non-keratinizing tumors showed 100% p53 positivity (p = 0.001). OSCC = oral squamous cell carcinoma

The immuno-expression of p53 was seen in 65.5% of cases (59/90). Regarding p53 expression across OSCC histological subtypes, no significant correlation was observed (p = 0.33). However, it was observed that well-differentiated SCC showed a weak staining pattern of p53 (Figure 3) and poorly differentiated SCC showed strong p53 expression (Figure 4). The expression of p53 increases from 39% in well-differentiated to 100% in poorly differentiated and non-keratinizing SCC (Figure 5), and this association with different histological grades was found to be significant, with a p-value of 0.001.

**Figure 6:**
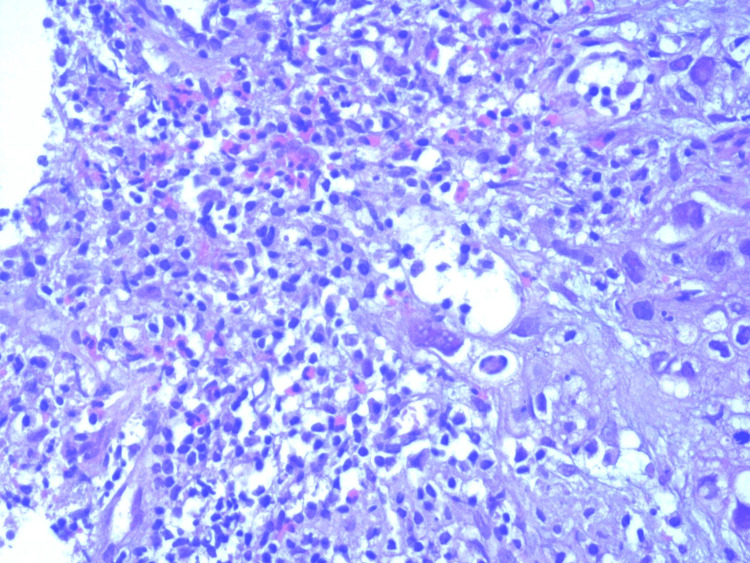
TATE gradient in well-differentiated OSCC (hematoxylin and eosin, 10× magnification). Prominent eosinophilic infiltration (TATE grade 3) is observed in the stroma of well-differentiated OSCC, associated with a favorable prognosis. OSCC = oral squamous cell carcinoma; TATE = tumor-associated tissue eosinophilia

**Figure 7:**
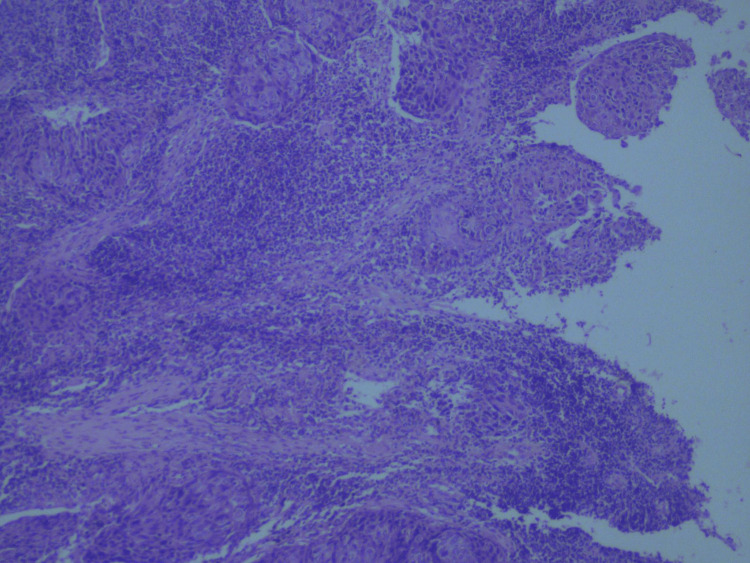
TATE gradient in poorly differentiated OSCC (hematoxylin and eosin, 10× magnification). Sparse eosinophilic infiltration (TATE Grade 1) is seen, correlating with higher tumor grade and poor differentiation. OSCC = oral squamous cell carcinoma; TATE = tumor-associated tissue eosinophilia

**Figure 8:**
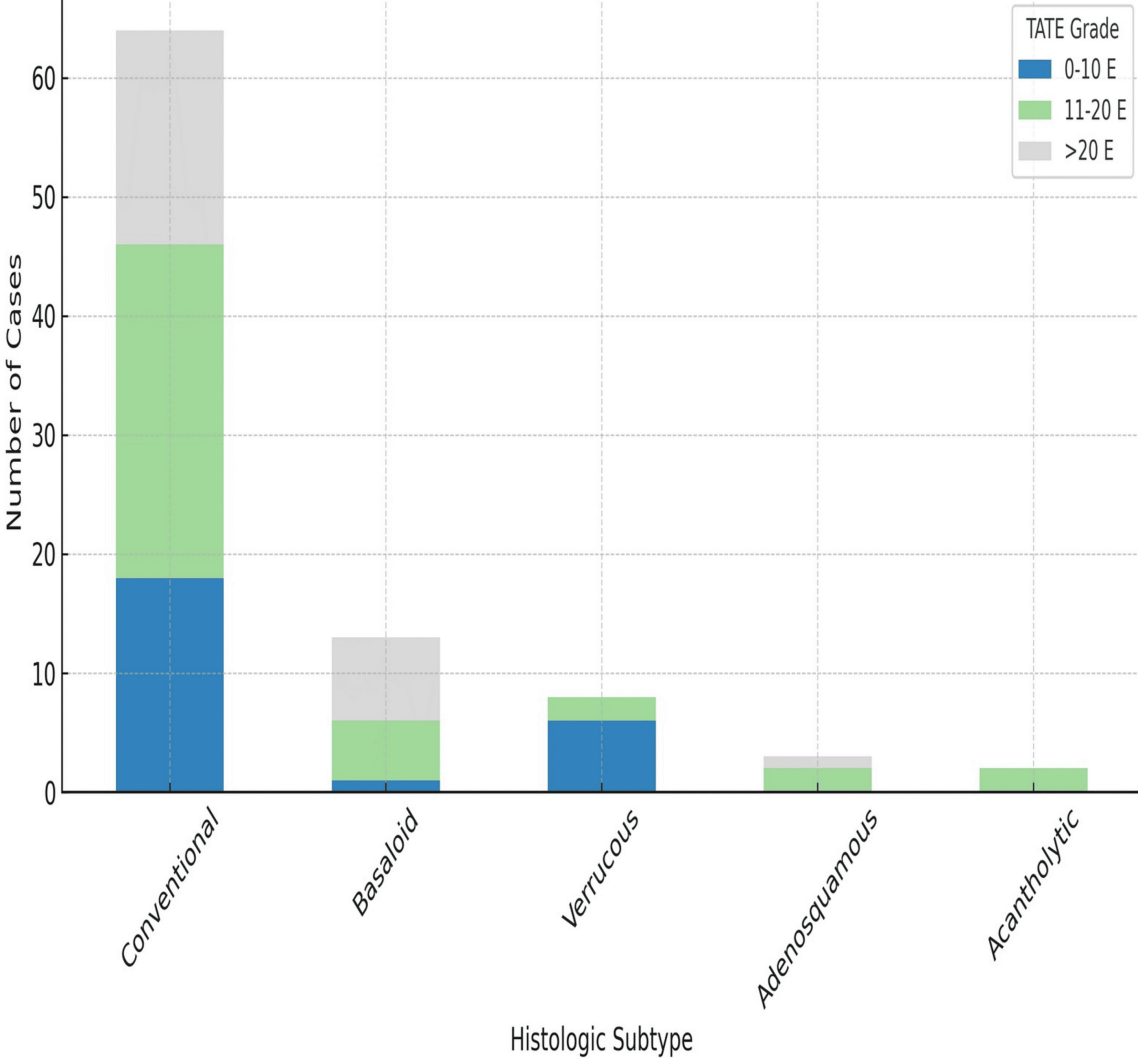
Association of TATE gradient with OSCC grades. This bar graph presents the distribution of TATE grades (0–10, 11–20, and >20 eosinophils per high-power field) across five histologic subtypes of OSCC: conventional, basaloid, verrucous, adenosquamous, and acantholytic. The data demonstrate significant variation in eosinophilic infiltration among different subtypes, with the conventional and basaloid variants showing higher proportions of grade 2 and 3 TATE. Statistical significance was established using the Pearson’s chi-square test (χ² = 17.980, p = 0.021). OSCC = oral squamous cell carcinoma; TATE = tumor-associated tissue eosinophilia; E = Eosinophils per high-power field

TATE gradient was also assessed, and its association with histological subtypes of OSCC was found to be significant (p = 0.02). This association was also statistically significant across different histological grades. TATE grade 3 was found in 51.2% of well-differentiated cancers, indicating that as TATE grade increases, the tumor becomes more differentiated (Figure 6). Most cases of poorly differentiated cancers had grade 1 eosinophilia; none had grade 3 eosinophilia, as depicted in Figure 7. A significant association was found between TATE gradient and different grades of SCC (Figure 8); hence, this parameter can be of prognostic significance, i.e., the higher the TATE grade, the better the prognosis.

To conclude, a statistically significant relation was observed between cyclin D1, p53, and TATE with different tumor grades, with p-values of 0.001, 0.001, and 0.003, respectively.

## Discussion

Our study revealed that cyclin D1 expression was observed in 61% (55/90) of OSCC cases. A statistically significant association was found between cyclin D1 expression and tumor grade (p = 0.001). Contrary to some previous studies, our results showed weaker expression of cyclin D1 in well-differentiated OSCC and markedly higher expression in poorly differentiated cases, suggesting that cyclin D1 expression increases with decreasing tumor differentiation. This may reflect its potential role in promoting aggressive tumor behavior.

A Sudanese study similarly found higher cyclin D1 expression in poorly differentiated SCC compared to well-differentiated cases, with a significant p-value of 0.0003) [6]. At Ayub Medical College, cyclin D1 expression was reported in 53.8% of OSCC cases and was significantly associated with higher grades, with more intense staining in poorly differentiated tumors, in contrast to older studies that showed stronger expression in lower grades [14].

Fatima et al. reported cyclin D1 overexpression in 77% of OSCC cases, with a significant correlation with higher grades (p = 0.003) [15]. Choudhary et al. found cyclin D1 overexpressed in 68% of cases and reported an inverse correlation between its expression and tumor differentiation (p = 0.02), aligning with our findings [11]. An Indian study also observed a significant association between cyclin D1 expression and higher histologic grades [16]. Likewise, Mishra et al. observed cyclin D1 positivity in 98% of OSCC cases, with stronger expression in poorly differentiated tumors, although this association was not statistically significant (p = 0.065) [17,18].

In our study, p53 expression was detected in 65.5% of OSCC cases. Its expression showed a stepwise increase with tumor grade: 39% in grade I, 80% in grade II, and 100% in grade III and non-keratinizing variants. This pattern showed a statistically significant association (p = 0.001). An Indian study similarly demonstrated p53 positivity in 58% of cases with a significant association with higher histologic grades [19], supported by another Indian cohort [20]. However, a Pakistani study by Zubair et al. reported increased p53 expression in OSCC but did not find a significant correlation with tumor grade [21].

TATE was also evaluated in our study and found to be significantly associated with tumor grade (p = 0.02). Grade 3 eosinophilia was predominantly observed in well-differentiated tumors and was absent in most poorly differentiated cases. This suggests that higher TATE is associated with better tumor differentiation and potentially a favorable prognosis. Siddique et al. also showed a significant association of TATE with OSCC histological grade [22], and findings from an Egyptian study further supported the correlation between TATE and tumor differentiation [23].

This was a single-center study with a relatively small sample size of 90 cases, limiting the generalizability of the findings. Potential confounders, including age, gender, tumor site, and risk factors, were recorded but not adjusted statistically, as the study had a descriptive, cross-sectional design. Moreover, the absence of long-term prognostic follow-up data restricts conclusions on clinical outcomes. Future research involving multicenter cohorts with follow-up is essential to establish the diagnostic and prognostic value of cyclin D1, p53, and TATE in OSCC.

## Conclusions

This study highlights the significant role of cyclin D1 and p53 expression in the progression of OSCC. Cyclin D1 and p53 expression showed a significant correlation with tumor grade. As OSCC advances to higher grades, a progressive increase in cyclin D1 and p53 expression and a decrease in TATE infiltration are noted. These findings emphasize the prognostic value of cyclin D1 and p53 as potential biomarkers in OSCC, aiding in tumor stratification and outcome prediction. The significant correlation of TATE with tumor differentiation further highlights its potential role in the tumor microenvironment and immune response. It also suggests a potential tumoricidal role of eosinophils, highlighting their importance in the tumor microenvironment. Given this association, TATE could serve as a convenient early prognosticator for head and neck cancers and should be routinely included in biopsy reports. Furthermore, assessing TATE may aid in treatment planning, particularly for malignancies predicted to follow an aggressive course. From a clinical perspective, understanding these molecular markers could help in refining therapeutic strategies, risk assessment, and patient monitoring. Future research should explore the functional implications of these biomarkers and their potential for targeted therapy, paving the way for personalized treatment approaches in OSCC management.
